# Alcohol use, depressive symptoms, and intimate partner violence perpetration: A longitudinal analysis among men with HIV in northern Vietnam

**DOI:** 10.1371/journal.pone.0240674

**Published:** 2020-10-16

**Authors:** Rebecca B. Hershow, H. Luz McNaughton Reyes, Tran Viet Ha, Geetanjali Chander, Nguyen Vu Tuyet Mai, Teerada Sripaipan, Constantine Frangakis, David W. Dowdy, Carl Latkin, Heidi E. Hutton, Audrey Pettifor, Suzanne Maman, Vivian F. Go

**Affiliations:** 1 Department of Health Behavior, Gillings School of Global Public Health, University of North Carolina at Chapel Hill, Chapel Hill, North Carolina, United States of America; 2 Yen Hoa Health Clinic, University of North Carolina Vietnam, Hanoi, Vietnam; 3 Department of Medicine, Johns Hopkins University School of Medicine, Baltimore, Maryland, United States of America; 4 Department of Epidemiology, Johns Hopkins University Bloomberg School of Public Health, Baltimore, Maryland, United States of America; 5 Department of Biostatistics, Johns Hopkins University Bloomberg School of Public Health, Baltimore, Maryland, United States of America; 6 Department of Health, Behavior and Society, Johns Hopkins University Bloomberg School of Public Health, Baltimore, Maryland, United States of America; 7 Department of Psychiatry and Behavioral Sciences, Johns Hopkins University School of Medicine, Baltimore, Maryland, United States of America; 8 Department of Epidemiology, Gillings School of Global Public Health, University of North Carolina at Chapel Hill, Chapel Hill, North Carolina, United States of America; USC Keck School of Medicine, Institute for Global Health, UNITED STATES

## Abstract

**Background:**

While the link between alcohol use and male-perpetrated intimate partner violence (IPV) has been well-established, research is needed to test whether psychosocial factors interact with alcohol use to exacerbate IPV perpetration. We tested whether depressive symptoms influenced the strength and/or direction of the alcohol-IPV relationship among men with HIV in Vietnam.

**Methods:**

This study is a secondary analysis using data from a randomized controlled trial conducted in Thai Nguyen, Vietnam. Participants were clinic patients with HIV and hazardous alcohol use. Questionnaires were administered at baseline, three, six, and 12 months. Alcohol use was assessed as proportion of days alcohol abstinent. Analyses were restricted to males who reported being married/cohabitating at baseline (N = 313). Multilevel growth models were used to test whether time-varying depressive symptoms modified the time-varying effect of alcohol use on IPV perpetration.

**Results:**

Time-varying depressive symptoms modified the effect of proportion of days alcohol abstinent on IPV perpetration. However, the pattern of effect modification was not as expected, as reporting depressive symptoms weakened the alcohol-IPV relationship. At times when participants screened negative for depressive symptoms, those who reported higher proportion of days alcohol abstinent than usual had significantly lower odds of IPV perpetration (Odds Ratio [OR] = 0.17, 95% Confidence Interval 0.06, 0.45, p = 0.0004). At times when participants screened positive for depressive symptoms, there was no observed effect of alcohol use on IPV perpetration (OR = 4.28, 95% CI 0.80, 22.78, p = 0.09).

**Conclusion:**

The findings highlight the complex nature of the alcohol-IPV relationship and the need to investigate the intersection between hazardous drinking, mental health, and IPV. Men who concurrently report depressive symptoms and heightened alcohol use may be socially isolated from an intimate partner or experiencing fatigue, leading to less alcohol-related IPV perpetration. Mental health interventions addressing depression and alcohol misuse integrated into HIV services may reduce IPV perpetration.

## Introduction

The link between alcohol use and male-perpetrated intimate partner violence (IPV) has been established [1–8]. The alcohol-IPV relationship is often explained by immediate alcohol intoxication effects, such as distorted perceptions of cues or lowered inhibitions, or by increased relationship conflict [4, 5, 7, 9–11]. However, meta-analytic reviews on alcohol use and IPV perpetration have observed large heterogeneity in effect sizes across studies [7–9, 12]. Research is needed to test whether psychosocial factors, such as depressive symptoms, influence the strength of the alcohol-IPV relationship [7–9, 12]. Such interactions may explain some of the heterogeneity found in the alcohol-IPV association, providing critical understanding on how to effectively intervene and reduce IPV perpetration among men. As both IPV and alcohol use lead to numerous health consequences, including HIV/STI infection, depression, physical injury, and death, IPV and alcohol interventions are urgently needed to improve the health and well-being of men and women at-risk for IPV [13–17].

Alcohol use and IPV perpetration are highly prevalent in Vietnam and largely shaped by sociocultural norms that stem from Confucianism [18]. Men are the key decision-makers and income earners of their households [19, 20]. Men's use of violence or aggression to exercise control is often seen as necessary to maintain a superior position [19, 20]. Studies estimate that 58% of married women reported ever experiencing IPV by their husbands [21] and 37% of men reported ever perpetrating IPV against their wife [22]. Men's alcohol use is also closely tied to their masculinity [23] and it is strongly encouraged through cultural practices and informal and formal social events [23–26]. The World Health Organization found that the annual per capita consumption of pure alcohol in Vietnam was 5.1 liters [14], which is greater than the global consumption of pure alcohol per capita (4.3 to 4.7 liters) [27]. Traditional gender norms characterize men as having volatile tempers and alcohol is perceived as a driver of men's aggressive or violent behavior [20]. Men’s use of violence under the influence of alcohol is often viewed as uncontrollable and an innate demonstration of masculinity [19, 20, 23].

The influence of depressive symptoms on the alcohol-IPV relationship is unexplored among men with HIV, a group at high risk for hazardous alcohol use, IPV perpetration, and forward HIV transmission [16, 17, 28–30]. In Vietnam, depressive symptoms are highly prevalent and a known correlate of IPV perpetration among men with HIV [31]. The link between depressive symptoms and IPV perpetration may be explained by self-control impairment, relationship conflict, or a bi-directional relationship between depressive symptoms and IPV perpetration [32–36]. However, there is a dearth of research in Vietnam and other global settings examining how depressive symptoms and alcohol use interact to influence IPV perpetration.

Alcohol use may be associated with IPV perpetration for some individuals, but not for others. Multiple threshold and disinhibition theories posit that each individual has an aggressive threshold determined by their propensity for aggression or by the extent to which their context encourages aggressive behavior [37–39]. These theories state that for each individual, IPV occurs when they surpass their aggressive threshold, meaning the strength of their aggressive motivations exceeds that of their inhibitions [37–39]. Depressive symptoms may interact with alcohol use to drive individuals past their aggressive thresholds. In particular, researchers have posited that depression may manifest as internalized anger and lead to self-control impairment or emotional dysregulation, making individuals who experience depressive symptoms more susceptible to the disinhibiting effects of alcohol intoxication on IPV perpetration [32–34].

Empirical evidence also suggests that the co-occurrence of alcohol use and mental health exacerbates violence outcomes. A nationally representative study in the United States found that reporting a severe mental illness did not predict violence, but reporting both a severe mental illness and substance abuse did [40]. Another United States study found that alcohol use strengthened the relationship between mental illness symptoms and perpetration of aggression [32]. In addition to these quantitative studies, qualitative research in India has suggested that depressive symptoms interact with alcohol use to drive IPV perpetration [41].

In this study, we tested whether depressive symptoms influenced the strength and/or direction of the alcohol-IPV relationship among men with HIV and hazardous alcohol use in Vietnam. We investigated whether the concurrent effect of alcohol use on IPV perpetration differed at times when an individual reported having depressive symptoms versus not having depressive symptoms. Our specific hypothesis was that the time-varying association between alcohol use and IPV perpetration would be stronger at times when an individual screens positive for depressive symptoms as compared to negative.

## Materials and methods

### Study design

This study is a secondary analysis of data from a three-arm randomized controlled trial (RCT) comparing the effects of two evidence-based, manually guided, individually delivered interventions to reduce alcohol use and determine the impact on viral load [42, 43]. From March 2016 to May 2018, data were collected from clinic patients with HIV and hazardous alcohol use (N = 440) in Thai Nguyen, a semi-urban province in northern Vietnam [42]. Thai Nguyen has the highest HIV prevalence among people who inject drugs (PWID; 34%) in Vietnam [44].

### Participant recruitment and data collection

Recruitment of study participants took place at seven antiretroviral treatment (ART) community clinics in Thai Nguyen. Recruitment was completed at one clinic before moving on to recruit potential participants at each subsequent clinic. Study interviewers introduced the project before administering written informed consent to those who were interested in participating in the baseline questionnaire. Those who provided consent completed an interviewer-administered screening survey. The survey included the World Health Organization (WHO) Alcohol Use Disorders Identification Test (AUDIT-C) survey items to determine eligibility for enrollment [45]. Inclusion criteria for eligibility included: (1) current patient on ART at the clinic; (2) AUDIT-C score ≥ 4 (for men) or ≥ 3 (for women) [45]; (3) 18 years of age or older; and (4) plan on residing in Thai Nguyen for the next 24 months. Exclusion criteria for eligibility included: (1) unable to participate in study activities due to psychological disturbance, cognitive impairment or threatening behavior (assessed by study staff); (2) unwilling to provide locator information; (3) unwilling to provide informed consent; and (4) currently participating in other HIV, drug use, or alcohol program, study, or intervention.

Eligible participants were asked to consent a second time to enroll in the study after interviewers explained the RCT study objectives, procedures, risks and benefits to the participants, and answered any questions. Consenting participants were assigned a unique identification number. They then completed the baseline questionnaire and Timeline Followback (TLFB) to measure daily alcohol use over the past 30 days [46]. Eligible participants were also randomly assigned to one of the three study arms in a 1:1:1 ratio.

At baseline, three, six, and 12 months, quantitative questionnaires and the TLFB were administered to all study participants. Each study visit lasted approximately two hours. Trained interviewers administered questionnaires through face-to-face interviews in a private room at the study site. Participants confirmed informed consent at each follow-up visit. The behavioral assessment collected quantitative data on sociodemographics, alcohol and drug use, mental health, and violence. At the end of each study visit, participants were reimbursed for their travel expenses and given 100,000 Vietnamese Dong (~$4.30 USD) as compensation for lost wages. Retention was 94% (405/430) at three months, 96% (410/427) at six months, and 94% (390/414) at 12 months excluding those who were incarcerated (n = 11/440; 2.5%) or died (n = 15/440; 3.4%) during the study period.

The study protocol received ethical approval from the Institutional Review Boards at the University of North Carolina Gillings School of Global Public Health, the Johns Hopkins University Bloomberg School of Public Health and the Thai Nguyen Center for Preventive Medicine. The study was registered at ClinicalTrials.gov (NCT02720237).

### Key measures

#### IPV perpetration

The widely used and validated six-item shortened Conflict Tactics Scale 2 (CTS2) was used to measure recent psychological, physical, and sexual IPV perpetration among any current or previous partner [47–49]. Recall periods varied based on the study visit (baseline: IPV perpetration in last 12 months; three-, six-, and 12-month follow-up: IPV perpetration in last three months). The numbers of recent IPV events reported over the study were skewed and zero inflation was observed in the data. As our sample was not large enough to assess IPV perpetration as a count variable, responses to all six items were categorized as those who reported recent IPV perpetration at least once and those who did not. Those who declined or did not know a response were classified as missing. A binary outcome variable was created for any form of recent IPV perpetration, defined as reporting recent psychological, physical, and/or sexual IPV perpetration [50].

#### Alcohol use

The TLFB was used to measure alcohol use; the tool has been shown to be valid and reliable in multiple settings [51, 52]. The TLFB is interviewer-administered and reconstructs a daily behavior calendar to help promote memory recall for alcohol use. Using the TLFB data, we assessed alcohol use as *proportion of days alcohol abstinent in past 30 days (0 to 1)*.

#### Depressive symptoms

Depressive symptoms were measured using the nine-item Patient Health Questionnaire-9 (PHQ-9) scale (Cronbach's alpha at baseline = 0.83) [53], a tool that has been validated in Vietnam [54]. A composite PHQ-9 score was calculated for each participant. Based on the standard scoring method used for the PHQ-9, those who scored zero to four were categorized as screening negative for depressive symptoms, and those who scored above four were categorized as screening positive for depressive symptoms [55]. A binary measure was also used as the distribution of PHQ-9 scores was highly skewed at baseline.

#### Covariates

Theoretical and empirical evidence was used to select variables with the potential to confound the relationship between alcohol use and IPV perpetration [1, 6, 56–60]. Exposure to violence as a child was measured using three items asking about ever witnessing interparental violence as a child, ever experiencing physical abuse as a child, or ever experiencing sexual abuse as a child. Responses were dichotomized; those who responded yes to any of the three questions were categorized as having been exposed to violence as a child. Involvement in community violence comprised two items asking about having ever been physically violent towards someone in their community or having ever experienced physical violence by someone in their community [61]. As both experiencing and perpetrating community violence increase the likelihood of IPV perpetration [1, 58, 62, 63], a composite variable was created for involvement in community violence.

Education, age, employment status, and injection drug use in the past three months were collected via self-report. Intervention arm was also selected as a covariate and was documented using a computer-generated randomization process.

### Data analysis

For this study, analysis was restricted to male participants who reported being married or cohabitating with a partner at baseline (N = 313) for two main reasons. First, a previous study conducted among men with HIV in northern Vietnam demonstrated that the alcohol-IPV relationship operates differently for men who were married or cohabitating versus those who were single, divorced, separated or widowed [31]. This finding is supported by studies in other settings showing that IPV prevalence and dynamics differ by relationship status [64, 65]. Second, a commonly used theoretical model in alcohol and IPV research posits that relationship conflict may explain the alcohol-IPV relationship [4, 5, 11]. Those who are married or cohabitating may have more opportunity for changes in relationship conflict during the study window as compared to those who are not.

Effect modification analyses were conducted in SAS 9.4 with multilevel growth models using maximum likelihood based on the Laplace estimation [66]. First, we determined the best-fitting unconditional growth model for IPV perpetration by testing linear and quadratic functional forms. The variance component for the random effect was removed from the model if it could not be estimated. The fixed effect was also removed from the model if it was non-significant.

All variables included in the analysis were centered [67]. The wave of data collection variable was re-coded to start at zero to ensure the intercept represents the log odds of IPV perpetration at baseline. The alcohol use variable used to estimate time-varying (or within-person) effects was person-mean centered. For depressive symptoms, the person means were calculated and included in the model as controls along with the dummy coded depressive symptoms variable. Age (years) was grand-mean centered and categorical covariates were dummy coded.

After determining the best-fitting unconditional growth model, the adjusted conditional growth model tested the time-varying effects of alcohol use and depressive symptoms and the two-way interactions among depressive symptoms and alcohol use on IPV perpetration adjusting for covariates. Any non-significant interactions were removed from the final model. If an interaction term was significant, the effect of alcohol use on the predicted probability of IPV perpetration was graphed at both levels of the depressive symptoms variable (0 or 1) to visualize the relationships. Standard errors (SEs) and p-values were generated to test the statistical significance of the intercepts and slopes represented in the graphs. Since participants were recruited from different ART community clinics, recruitment site was initially tested as a fixed effect to assess if it influenced results. As it did not change the results, it was removed to make models more parsimonious.

## Results

### Descriptive statistics

The sample included 313 male participants who were married or cohabitating at baseline (Mean age: 40.8 years, Standard deviation [SD] = 5.6; Table 1). The median percent days alcohol abstinent increased over time (Baseline: 36.7, Interquartile range [IQR] = 66.7; 12-month follow-up: 76.7, IQR = 66.7). The proportion of participants who screened positive for depressive symptoms decreased over time (Baseline: N = 68/313, 21.7%; 12-month follow-up: N = 43/279, 15.4%).

**Table 1:** Characteristics of male participants who were married or cohabiting with a partner (N = 313).

	**Baseline (N = 313)**	**3 months (N = 287)**	**6 months (N = 292)**	**12 months (N = 279)**
	N (%)			
**Sociodemographic Characteristics**				
Mean age in years (SD)	40.8 (5.6)			
Highest level of education				
Technical training/College or university or less	52 (16.6)			
High school or less	189 (60.4)			
Secondary school or less	48 (15.3)			
Primary school or less	24 (7.7)			
Employment status				
Employed full- or part-time	262 (83.7)	243 (84.7)	255 (87.3)	245 (87.8)
Unemployed/Retired	51 (16.3)	44 (15.3)	37 (12.7)	34 (12.2)
Virally suppressed^a^^,^^b^	268 (85.6)	247 (86.4)	257 (88.0)	237 (84.9)
**Alcohol Use**				
Median percent days abstinent in past 30 days (IQR)	36.7 (66.7)	70.0 (60.0)	76.7 (73.3)	76.7 (66.7)
**Psychosocial Factors**				
Depressive symptoms in past 2 weeks	68 (21.7)	48 (16.7)	49 (16.8)	43 (15.4)
Used injection drugs in past 3 months^b^	69 (22.0)	60 (21.0)	64 (21.9)	57 (20.4)
Exposed to violence as a child^c^	114 (36.4)			
Ever involved in community violence^b^	135 (43.1)	110 (38.5)	119 (40.8)	139 (49.8)

Abbreviations: SD = Standard deviation; IQR = Interquartile range.

^a^Achieving viral suppression was defined as having less than 20 copies/ml.

^b^Missing data due to refused to answer or don’t know: Viral suppression at three months: N = 1; Injection drug use at three months: N = 1; Ever involved in community violence at three months: N = 1.

^c^Exposed to violence as a child includes having ever witnessed interparental violence as a child or experienced physical or sexual abuse as a child.

**Fig 1:**
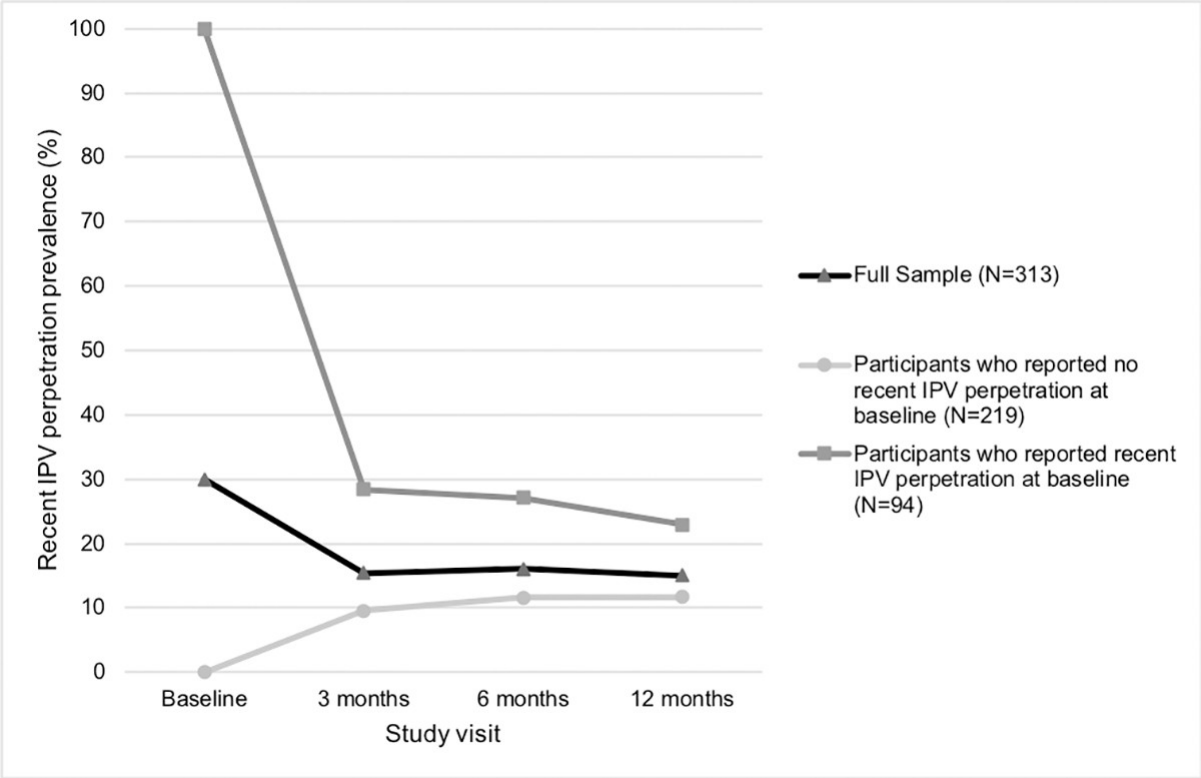
Prevalence of recent intimate partner violence perpetration among the full sample (N = 313), among those who reported no recent IPV perpetration at baseline (N = 219), and among those who reported recent IPV perpetration at baseline (N = 94). Abbreviations: IPV = Intimate partner violence.

The prevalence of any form of IPV perpetration in the past 12 months was 30.0% (94/313) at baseline (Table 2). At three-month follow-up, the prevalence of IPV in the past three months was 15.4% (44/286), and last-three-month IPV prevalence remained fairly stable throughout the remainder of the study period (six-month follow-up: N = 47/292, 16.1%; 12-month follow-up: N = 42/279, 15.1%). Participants who reported recent IPV perpetration at baseline (N = 94/313) had higher IPV perpetration prevalence estimates across all subsequent study visits as compared to participants who reported no recent IPV perpetration at baseline (N = 219/313; Fig 1).

**Table 2:** Prevalence of intimate partner violence perpetration across study visits^a^^,^^b^.

	**Baseline (N = 313)**	**3 months (N = 287)**	**6 months (N = 292)**	**12 months (N = 279)**
	N (%)			
**Recent psychological IPV perpetration^c^**				
No	231 (73.8)	250 (87.4)	252 (86.3)	242 (86.7)
Yes	82 (26.2)	36 (12.6)	40 (13.7)	37 (13.3)
**Recent physical IPV perpetration**				
No	298 (95.2)	282 (98.3)	285 (97.6)	275 (98.6)
Yes	15 (4.8)	5 (1.7)	7 (2.4)	4 (1.4)
**Recent sexual IPV perpetration**				
No	293 (93.6)	276 (96.2)	285 (97.6)	270 (96.8)
Yes	20 (6.4)	11 (3.8)	7 (2.4)	9 (3.2)
**Recent physical/sexual IPV perpetration**				
No	280 (89.5)	272 (94.8)	279 (95.5)	266 (95.3)
Yes	33 (10.5)	15 (5.2)	13 (4.5)	13 (4.7)
**Any form of recent IPV perpetration^c^**				
No	219 (70.0)	242 (84.6)	245 (83.9)	237 (84.9)
Yes	94 (30.0)	44 (15.4)	47 (16.1)	42 (15.1)

Abbreviations: IPV = Intimate partner violence.

^a^Percentages may not sum to 100 due to rounding.

^b^At baseline, participants were asked about IPV perpetration in the past 12 months; at subsequent follow-up study visits, participants were asked about IPV perpetration in the past three months.

^c^Missing data due to refused to answer or don’t know: Recent psychological IPV perpetration at three months: N = 1; Any form of recent IPV perpetration at three months: N = 1.

### Unconditional growth model

The best fitting unconditional growth model for IPV perpetration comprised a random intercept with linear and fixed effects for time. The odds of IPV perpetration significantly decreased over time (OR = 0.45, 95% CI 0.34, 0.59, p<0.0001). At baseline, the variance in IPV perpetration significantly differed between participants (Intercept: b = -1.09, SE = 0.14, p<0.0001).

### Adjusted analyses

**Fig 2:**
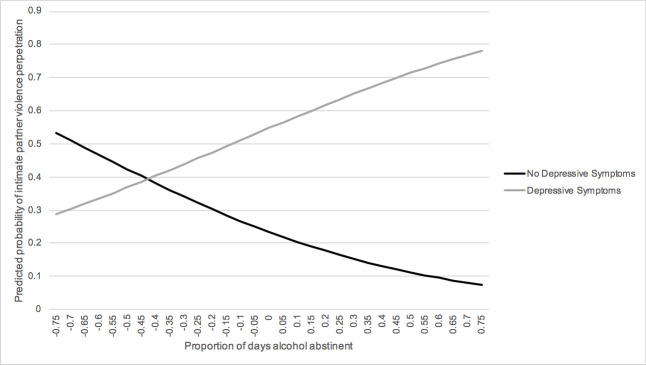
Estimated intimate partner violence perpetration as a function of depressive symptoms and proportion of days alcohol abstinent at times when participants screened positive and negative for depressive symptoms. On the X-axis, the zero value is each participant's average proportion of days alcohol abstinent reported across the study period. Values greater than zero represent participants reporting higher proportion of days alcohol abstinent than average and values less than zero represent participants reporting lower proportion of days alcohol abstinent than average.

Depressive symptoms significantly modified the effect of proportion of days alcohol abstinent on IPV perpetration (Table 3). However, the pattern of effect modification did not support our hypothesis, as having depressive symptoms was found to weaken the inverse relationship between proportion of days alcohol abstinent and IPV perpetration (Fig 2). At times when individuals screened negative for depressive symptoms, those who reported higher proportion of days alcohol abstinent than usual were less likely to report IPV perpetration (Slope: OR = 0.17, 95% CI 0.06, 0.45, p = 0.0004; Table 4). In contrast, at times when individuals screened positive for depressive symptoms, there was no significant association between proportion of days alcohol abstinent and IPV perpetration (Slope: OR = 4.28, 95% CI 0.80, 22.78, p = 0.09).

**Table 3:** Final adjusted conditional growth model^a^ (N = 313).

	**aOR (95% CI)**	**p-value**
**Fixed Effects**		
Intercept	0.30 (0.13, 0.71)	0.006
Time	0.55 (0.41, 0.73)	<0.0001
Alcohol use	0.17 (0.06, 0.45)	0.0004
Depressive symptoms	3.96 (2.18, 7.20)	<0.0001
Alcohol use*Depressive symptoms	25.02 (3.54, 176.78)	0.001
**Random Effects**		
***Variance Components***		
Covariance	-0.0001 (0.25)	
Variance	0.53 (0.57)	

Abbreviations: aOR = Adjusted odds ratio; CI = Confidence Interval.

^a^Controlling for education, grand-mean centered age, exposure to violence as a child, employment status, injection drug use, involvement in community violence ever, and intervention arm.

**Table 4:** Simple intercepts and slopes, proportion of days alcohol abstinent by depressive symptoms.

	Intercept		Slope	
	OR (95% CI)	p-value	OR (95% CI)	p-value
**No Depressive Symptoms**	0.30 (0.13, 0.70)	0.006	0.17 (0.06, 0.45)	0.0004
**Depressive Symptoms**	1.21 (0.44, 3.32)	0.72	4.28 (0.80, 22.78)	0.09

Abbreviations: OR = Odds Ratio; CI = Confidence Interval.

## Discussion

In this study, alcohol use and depressive symptoms did not act synergistically to increase the odds of IPV perpetration. At times when participants reported having depressive symptoms, the effect of alcohol use on IPV perpetration became non-significant. However, at times when participants reported having no depressive symptoms, more alcohol use was associated with significantly higher odds of IPV perpetration. This finding diverges from empirical and theoretical evidence suggesting that alcohol use and depression interact to exacerbate violence outcomes [32, 37–41, 68]. Previous research among Vietnamese men with HIV identified depressive symptoms and alcohol use as independent correlates of IPV perpetration [31]. Our study builds on these findings to suggest that while both depressive symptoms and alcohol use may be independently associated with IPV perpetration [31], they may not jointly increase odds of IPV perpetration.

Our study findings do not align with the multiple threshold and disinhibition theories, which suggest that people who use alcohol and experience depressive symptoms may have higher odds of IPV perpetration as compared to those who only use alcohol [37–39]. However, an inherent assumption in these theories is that alcohol use and depressive symptoms are aggression-provoking factors both independently and jointly [37–39]. Our study suggests that Vietnamese men with HIV who are concurrently using alcohol and experiencing depressive symptoms may not express aggression-provoking symptoms, such as hostility or anger [33, 69]. The combination of alcohol use, depressive symptoms, and HIV infection may lead to decreased energy and fatigue [70, 71], resulting in no relationship between alcohol use and IPV perpetration. Additionally, the co-occurrence of alcohol and depressive symptoms may result in other forms of violence or aggression beyond IPV perpetration; this should be tested in future research.

Another potential explanation may be the unique intersection between depressive symptoms, alcohol use, and social isolation in Vietnam. Men may respond to experiences of depression with social isolation [72, 73], leading to reduced alcohol use as drinking alone is uncommon [23, 24]. Instead, drinking centers around social interactions in Vietnam [23, 24, 74]. Those experiencing the co-occurrence of heightened alcohol use and depressive symptoms may represent a particularly isolated group that is not engaging with their partner, leaving limited opportunity for alcohol-related IPV events to occur. Research is needed to understand the circumstances under which men with HIV may or may not use alcohol as a coping strategy when experiencing depressive symptoms.

Due to the high prevalence of hazardous drinking and depressive symptoms among men with HIV globally and in Vietnam [71, 74], intervening on alcohol use and depression may be promising IPV prevention strategies. A quarter of Vietnamese men with HIV screened positive for depressive symptoms and nearly half (46%) screened positive for hazardous drinking [31]. Incorporating mental health services addressing depression and alcohol misuse into HIV care and treatment may reduce depression, alcohol use, and IPV perpetration among men with HIV. Systematic reviews have found that mental health treatments for common mental disorders and for alcohol misuse could effectively reduce IPV [75, 76]. However, there is limited research on the impact of mental health treatment targeting depression on IPV perpetration instead of IPV victimization [75]. Further, there is limited evidence demonstrating the effectiveness of mental health interventions on IPV perpetration in low- and middle-income settings [75, 76].

A large proportion of the participants currently or previously injected drugs because the HIV epidemic in Vietnam is largely driven by injection drug use [44, 77]. PWID with HIV have a higher risk of mortality than non-PWID with HIV [78] and hazardous alcohol use and depressive symptoms are highly prevalent among PWID with HIV [74, 79]. As the co-occurrence and dynamics of depressive symptoms and alcohol use may differ between HIV-infected men with and without a history of injection drug use, generalizability of study findings should be limited to men with HIV in countries with injection-driven HIV epidemics. Additionally, it is difficult to compare IPV prevalence estimates from our study to other Vietnam studies as the IPV measures and recall periods often differ [22, 49, 80].

We are not able to characterize the gender of all participants' partners due to missing data (N = 42/313). All participants with available data reported recently having female sexual partners only and having female main partners. Additionally, we did not collect data on the length of participants' intimate relationships. Participants whose relationships ended during the study may have been less likely to report IPV perpetration after their relationships ended. However, the vast majority of participants reported stable relationship statuses across study visits.

Variables measured using self-report may have been biased due to social desirability, resulting in more conservative estimates of IPV perpetration and depressive symptoms [81, 82]. This is probable as the questionnaires were interviewer-administered, which may have made participants less likely to report depressive symptoms or IPV perpetration [53]. However, interviewers received extensive training on questionnaire administration, reducing the likelihood of social desirability bias. Estimates of alcohol use may also be subject to recall bias as data were collected via self-report. However, sub-analyses of the study data validated the self-report alcohol measures using phosphatidylethanol (PEth) [42], a biomarker that is a direct metabolite of alcohol consumption [83].

Participants who dropped out of the study may have been more likely to perpetrate IPV than those who were retained in the study, resulting in an underestimation of IPV perpetration. We also used a binary measure of depressive symptoms in analyses, which may have led to a loss of meaningful information and attenuated effects.

Despite these limitations, this longitudinal study contributes important findings on the intersection between hazardous alcohol use, depressive symptoms, and IPV perpetration in a high-risk, understudied population. Unexpectedly, having depressive symptoms was found to weaken the alcohol-IPV relationship. These results suggest that men who reported higher alcohol use and depressive symptoms than usual may be experiencing extreme social isolation from an intimate partner, leading to less opportunity for alcohol-related IPV perpetration. Alternatively, men with HIV who are concurrently using alcohol and experiencing depressive symptoms may have fatigue or reduced energy, decreasing risk of alcohol-related IPV perpetration. Previous mental health interventions aiming to reduce IPV perpetration have either targeted participants who misuse alcohol or those who screen positive for depressive symptoms [75]. This study suggests that mental health interventions for men with HIV should target both groups—those who screen positive for hazardous drinking or depressive symptoms—to effectively reduce IPV perpetration.
